# Enhanced interhemispheric functional connectivity in patients with functional anorectal pain

**DOI:** 10.1038/s41598-025-26490-3

**Published:** 2025-11-27

**Authors:** Xiangjun Xu, Wenju Pei, Shubo Gao, Shuai Wang, Mingfeng Fan, Hao Yu, Churan Sun, Yu Wan, Cong Zhou, Yang Jiao

**Affiliations:** 1 Department of Anorectal Surgery, Affiliated Hospital of Jining Medical University, Jining, China; 2 Department of Radiology, Affiliated Hospital of Jining Medical University, Jining, China; 3 School of Mental Health, Jining Medical University, Jining, China; 4 Department of Psychology, Affiliated Hospital of Jining Medical University, Jining Medical University Clinical College, Jining, China

**Keywords:** Functional anorectal pain, Functional magnetic resonance imaging, Functional connectivity, Voxel-mirrored homotopic connectivity, Pain, Neuroimaging, Biomarkers, Neurology, Neuroscience

## Abstract

Functional anorectal pain (FAP) is a chronic condition with unclear pathophysiological mechanisms that is often linked to psychological distress. This resting-state functional magnetic resonance imaging (rs-fMRI) study investigated aberrant interhemispheric connections in 30 FAP patients versus 21 matched healthy controls (HC) via seed-based functional connectivity (FC) and voxel-mirrored homotopic connectivity (VMHC). Compared with HC, FAP patients presented enhanced FC between the left middle frontal gyrus (MFG.L) and regions such as the right MFG (MFG.R) and left superior temporal gyrus (STG.L). VMHC analysis revealed increased patterns in the MFG.L and left superior medial frontal gyrus (SFGmed.L) in FAP patients. Moreover, the strength of FC between the MFG.L and MFG.R was negatively correlated with age, indicating that this heightened connection may diminish with age. These findings indicate that FAP involves aberrant interhemispheric hyperconnectivity, which may play crucial roles in pain perception and emotional processing. The age-dependent decline in FC highlights the eroding of neuroplasticity in aging patients. These neural alterations could serve as diagnostic biomarkers and provide targets for therapeutic interventions. Our work positions FAP within a brain-gut axis dysregulation framework and suggests circuit-specific therapeutics to restore neural homeostasis.

**Supplementary Information:**

The online version contains supplementary material available at 10.1038/s41598-025-26490-3.

## Introduction

Functional anorectal pain (FAP) is a chronic pain condition characterized by persistent discomfort in the anorectal region, with no clear organic or structural findings upon standard clinical examination such as anoscopy and palpation^1^. Despite its prevalence, the pathophysiological mechanisms underlying FAP remain largely unclear. This condition is often associated with significant psychological distress, including symptoms of depression and anxiety, which can further exacerbate the pain experience^2,3^. Although direct evidence in FAP is evolving, features of personality disorders or maladaptive personality traits may also be present in some patients, potentially influencing clinical presentation and treatment response^4–7^. The pain symptoms can be significantly alleviated by paroxetine, as identified in our previous findings^3^. The complex interplay between physical symptoms and psychological factors suggests that FAP is not solely a result of peripheral abnormalities but also involves central nervous system processes. Recent research has indicated that FAP is closely associated with neurobiological factors, such as alterations in brain structure and function, as well as psychological factors, including mood disorders and cognitive dysfunction, particularly in domains such as executive function, attention, and memory^8^. Understanding these multifactorial contributors is essential for developing effective treatment strategies and improving the quality of life for patients with FAP.

Advancements in neuroimaging techniques, particularly resting-state functional magnetic resonance imaging (rs-fMRI), have provided valuable insights into the brain alterations associated with chronic pain conditions^9,10^. Research utilizing fMRI has consistently suggested that regions such as the superior frontal gyrus (SFG), orbitofrontal cortex (OFC), middle frontal gyrus (MFG), and cerebellum display notable changes in chronic pain patients^11–13^. These brain areas play key roles in pain processing, emotion regulation, and cognitive control^12,14–18^. Previous research has also shown that altered functional connectivity (FC) in these regions is associated with chronic pain conditions, suggesting a potential link between inter-regional functional connections and pain perception^10^. Kim et al. reported that fibromyalgia patients have altered structural covariance networks involving the dorsolateral prefrontal cortex (DLPFC) and cerebellum, which are associated with heightened pain sensitivity^19^. In addition, the cerebellum receives nociceptive input and is activated during both acute and chronic pain^20^. The connectivity between the cerebellum and other brain regions indicates its important role in integrative functions related to pain perception and emotional responses^21^.

Our previous multimodal MRI investigations of FAP^8^ utilized structural magnetic resonance imaging (sMRI), diffusion tensor imaging (DTI), and rs-fMRI to investigate brain alterations in FAP patients, revealing complex alterations in gray matter regions such as the superior frontal gyrus (SFG), fusiform gyrus, middle occipital gyrus, and compromised white matter integrity in the superior longitudinal fasciculus, forceps minor, and anterior thalamic radiation, as well as increased neural activity in the right cerebellum and right MFG (MFG.R). These findings indicate that FAP is linked to extensive brain changes, with certain regions possibly being key contributors to symptoms. Given the prominent functional alterations observed in the right hemisphere, we hypothesized that communication and coordination between the hemispheres might be disrupted. Although lateralized activity suggests an imbalance, it does not fully capture the functional integration between homotopic regions. Voxel-mirrored homotopic connectivity (VMHC) measures the correlation between blood-oxygen-level-dependent time series of symmetrical brain regions, thereby reflecting interhemispheric information exchange and integration. This method provides a direct assessment of interhemispheric synchrony, allowing us to distinguish whether the observed lateralization co-occurs with pathological hypersynchrony, which may reflect impaired inhibition, or hyposynchrony, suggestive of functional disconnection. As such, VMHC offers a novel and necessary level of insight that extends beyond what can be inferred from regional activation changes alone^22^. As a rs-fMRI method for analyzing interhemispheric functional connections, it is vital for studying brain information integration and has been widely used to assess interhemispheric homotopic connectivity in various neuropsychiatric disorders^23–25^ and pain research^26,27^. This research direction is crucial for understanding whether the observed right-hemisphere-predominant changes reflect a broader pattern of brain function lateralization in FAP patients. By exploring interhemispheric homotopic connectivity, we can determine whether there is an imbalance in communication between brain hemispheres and how it might contribute to the pathophysiology of FAP.

Given the complex interplay between pain perception, emotional distress, and cognitive control in FAP, it is essential to investigate the functional patterns in these regions to better understand the neural mechanisms underlying this condition. This study aims to explore the imbalanced resting-state FC (rs-FC) in FAP patients, focusing on the MFG, cerebellum, and SFG, and to determine how these changes correlate with pain symptoms. Additionally, this research explores interhemispheric homotopic connectivity with the VMHC approach. By elucidating the above issues in FAP, we hope to provide new insights into the pathophysiology of this condition and identify potential targets for therapeutic interventions.

## Materials and methods

### Participants

The study was conducted in accordance with the principles of the Helsinki Declaration, and approval was obtained from the Research Ethics Committee of the Affiliated Hospital of Jining Medical University. A total of 30 patients diagnosed with FAP and 21 healthy controls (HC) were recruited from the Department of Anorectal Surgery and the Health Management Center of the Affiliated Hospital of Jining Medical University. All participants provided informed consent and underwent comprehensive medical evaluations, including medical history review, physical examination, and blood biochemical analysis. FAP patients, aged between 18 and 65 years, were diagnosed and classified on the basis of the Rome IV criteria for disorders of gut-brain interaction (DGBI) by experienced colorectal specialists. The exclusion criteria were severe physical illnesses that prevented participation, current or past mental/neurological disorders or brain trauma, previous anal or rectal surgeries, distinct secondary anal or rectal pain, severe gastrointestinal conditions, medications affecting gastrointestinal function, a history of substance abuse or dependence, pregnancy or lactation, and MRI contraindications such as heart stents, pacemakers, or metal dentures. Healthy controls were matched to FAP patients in terms of age, sex, and exclusion criteria, with all the subjects being right-handed.

### Clinical data collection

All the FAP subjects were asked to complete a clinical data questionnaire before undergoing MRI scanning. The questionnaire collected information on disease progression and used the short-form McGill Pain Questionnaire (SF-MPQ) for pain assessment. The SF-MPQ includes 11 pain - intensity assessments (PRI: S), 4 pain emotion items (PRI: A), a Visual Analogue Scale (VAS), and Present Pain Intensity (PPI) to measure total pain intensity. Moreover, all subjects underwent assessment of depressive and anxiety symptoms and sleep quality by completing the Hamilton Depression Scale (HAMD), Hamilton Anxiety Scale (HAMA), and Pittsburgh Sleep Quality Index (PSQI) under psychiatrist guidance.

### MRI data acquisition

MRI data were collected using the Philips Ingenia CX 3.0T MRI scanner. Participants were positioned supine with their heads secured in an advanced position on the examination table. Sponge pads were placed symmetrically on both sides of the head to minimize movement. Before the scan, the participants were instructed to relax their entire body, keep their eyes closed but remain awake, avoid falling asleep, and refrain from engaging in specific mental activities. After the scan, participants were asked whether they had fallen asleep during the process. High-resolution T1-weighted images were obtained using three-dimensional Brain Volume Imaging (3D-TFE) with the following parameters: TR/TE = 6.6 ms/3.0 ms, FOV = 256 mm × 256 mm, slice thickness = 1.0 mm, no gap, voxel size = 1 mm × 1 mm × 1 mm, flip angle = 12°, scan matrix = 256 × 256, pixel bandwidth = 271 Hz, number of slices = 180, and scan time = 4 min and 20 s. Diffusion-weighted imaging (DWI) was performed using a single-shot echo planar imaging (EPI) sequence with the following parameters: TR/TE = 3800 ms/76 ms, FOV = 256 mm × 256 mm, acquisition matrix = 128 × 128, slice thickness = 2 mm, no gap, and a total of 62 slices covering the entire brain. Each participant underwent 64 non-collinear diffusion-weighted scans (b = 1000 s/mm²) and one unweighted (b = 0 s/mm²) scan. The acquisition of DWI data was part of the overarching multimodal imaging protocol for this cohort. The analysis of white matter integrity from this DWI data has been reported previously^8^. The present study focuses specifically on the analysis of rs-fMRI. Rs-fMRI data were acquired using a gradient-echo planar imaging sequence with the following parameters: TR/TE = 2000 ms/30 ms, FOV = 220 mm × 220 mm, slice thickness/gap = 3.0 mm/1.0 mm, scan matrix = 80 × 80, voxel size = 2.75 mm × 2.75 mm × 3 mm, number of slices = 36, and flip angle = 65°. The scan duration for each rs-fMRI session was 6 min and 12 s, with a total of 180 time points acquired. Additionally, routine MRI scans including T1-weighted imaging (T1WI), T2-weighted imaging (T2WI), and T2-fluid-attenuated inversion recovery (FLAIR) were performed to exclude participants with brain structural abnormalities.

### MRI data processing

MRI data were processed and analyzed via the GRETNA toolbox v2.0.0^28^ in Matlab 2018a (MathWorks, Natick, MA, USA). The preprocessing pipeline included discarding the first five time points, followed by slice-timing correction, head motion correction, spatial normalization to a standard space with 3 × 3 × 3 mm^3^ voxel dimensions using the structural image, spatial smoothing with a 6 mm full-width at half-maximum Gaussian kernel, removal of linear trends, regression of 24 Friston head motion parameters and white matter and cerebrospinal fluid signals, and application of a [0.01, 0.08] Hz temporal bandpass filter. For FC analysis, six predefined ROIs were selected a priori based on regions identified as structurally or functionally altered in our previous study of this cohort^8^. The seeds included the left SFG (SFG.L) [x = − 18, y = 8, z = 71], right SFG (SFG.R) [x = 18, y = 8, z = 71], left cerebellum [x = − 15, y = − 66, z = − 15], right cerebellum [x = 15, y = − 66, z = − 15], left MFG (MFG.L) [x = − 39, y = 51, z = 21], MFG.R [x = 39, y = 51, z = 21], were selected as the seed regions. Spherical ROIs with an 8 mm diameter were created for each seed region, and Pearson correlation coefficients were calculated between the average signal of each ROI and the signals of all the brain voxels. Fisher Z-transformation was applied to the correlation coefficients for statistical analysis, which was restricted to gray matter regions defined by the Automated Anatomical Labeling (AAL) atlas (116 regions). The results were visualized by using the BrainNet Viewer toolbox^29^.

VMHC analysis was performed using the right hemisphere mask from the AAL atlas applied to the preprocessed functional data. To ensure exact voxel-wise correspondence, all functional images were spatially normalized to a symmetric MNI template prior to the analysis. This was achieved by creating a study-specific symmetric template from the normalized T1 images of all participants and then registering both structural and functional images to this template. This method calculates the FC between symmetric voxels, reflecting the symmetry of FC between the brain hemispheres. The VMHC map was generated by calculating the *Pearson’s* correlation coefficient between the residual time series of each voxel and its mirrored counterpart in the opposite hemisphere, with lower VMHC values indicating higher asymmetry in functional activity. The resulting correlation coefficients underwent Fisher *z*-transformation to enhance normality, yielding the VMHC values for subsequent statistical analysis.

### Statistical analysis

Statistical analysis was performed using SPSS version 21.0. Independent samples *t*-tests were used to compare demographic data, clinical symptoms, and psychological scale scores between FAP patients and HC. The chi-square test was used to compare the distribution of sex assigned at birth between the two groups. All tests were two-tailed, with statistical significance set at *P* < 0.05.

Statistical analysis was performed using the Statistical Parametric Mapping (SPM) version 12 (Release 7771, released on January 13, 2020). Two-sample *t*-tests were applied to compare the FC and VMHC differences between FAP patients and HC, with sex assigned at birth and age as covariates of no interest to isolate the group effect. For both the FC and VMHC results, statistical significance was determined using Alphasim correction for multiple comparisons, which provides a well-established and validated balance between statistical sensitivity and Type I error control for fMRI data analysis. The voxel-wise threshold was *P* < 0.001, and clusters with more than 10 voxels were considered significant.

Once statistically significant differences in imaging indices were observed between FAP patients and HC, post-hoc correlation analyses were conducted to explore the relationships between these indices and clinical variables. These exploratory correlations were performed using Pearson’s correlation without including additional covariates. All clinical correlation analyses were restricted to the FAP patient group, as the relevant clinical data (e.g., pain measures) were not collected from the HC. The threshold for statistical significance was set at *P* < 0.05.

## Results

### Clinical data results

No statistically significant differences were found in terms of age and sex distribution between FAP patients and HC. Compared with HC, FAP patients showed significantly higher HAMD, HAMA, and PSQI scores (*P* < 0.001). The detailed demographic data is presented in Table 1.

**Table 1:** Demographic clinical and characteristics of functional anorectal pain patients and healthy controls.

	FAP(*n* = 30)	HC(*n* = 21)	T/χ^2^	*P*
Age (year)	51.20 ± 8.48	50.14 ± 8.43	0.44	0.662
Sex assigned at birth (male/female)	7/23	2/19	1.62	0.203
Duration (month)	6 (6, 12)	–	–	–
SF-MPQ	9.17 ± 2.78	–	–	–
PRI: S	2.33 ± 1.06	–	–	–
PRI: A	2.20 ± 1.13	–	–	–
VAS	2.10 ± 0.76	–	–	–
PPI	2.47 ± 0.73	–	–	–
HAMD	9.33 ± 7.39	1.19 ± 2.36	4.87	< 0.001**
HAMA	9.80 ± 7.89	0.76 ± 1.00	5.20	< 0.001**
PSQI	8.47 ± 5.03	3.76 ± 1.59	4.22	< 0.001**

Clinical pain measures (SF-MPQ) were not administered to HC following a screening process that confirmed the absence of pain symptoms, as these measures are not applicable to asymptomatic individuals.

FAP, Functional anorectal pain; HAMA, Hamilton Anxiety Scale; HAMD, Hamilton Depression Scale; HC, healthy controls; PPI: Present Pain Intensity; PRI: A: Pain Rating Index - Affective; PRI: S: Pain Rating Index - Sensory; PSQI, Pittsburgh Sleep Quality Index; SF-MPQ, short-form of McGill pain questionnaire; VAS: Visual Analogue Scale.

**: *P* < 0.001.

### Seed-based FC between groups

**Fig. 1:**
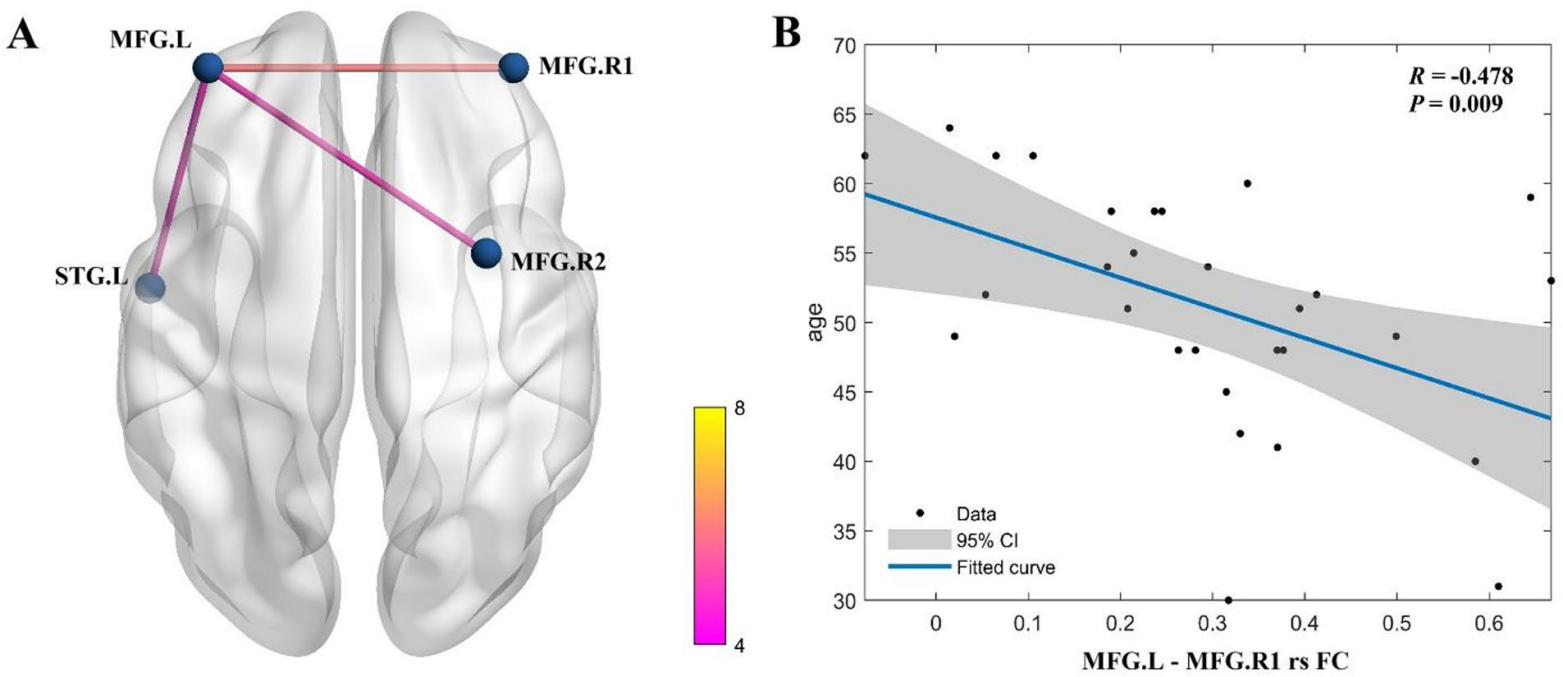
Altered functional connectivity (FC) in functional anorectal pain (FAP). (**A**) Increased functional connectivity in FAP patients versus healthy controls (HC). Significant clusters (Alphasim-corrected *P* < 0.001) show enhanced connectivity between the left middle frontal gyrus (MFG.L; MNI: − 39, 51, 21) and two regions of the right middle frontal gyrus (MFG.R; MNI: 45, 54, 18, and MNI: 33, 3, 54), and between MFG.L and left superior temporal gyrus (STG.L; MNI: − 54, − 6, − 3). (**B**) Negative correlation between MFG.L-MFG.R connectivity strength and age in FAP patients (*R* = − 0.478, *P* = 0.009).

increased FC was observed between the MFG.L and three other brain regions, including FC between the MFG.L and two MFG.R regions, and one FC between the MFG.L and the left superior temporal gyrus (STG.L), with Alphasim-corrected *P* < 0.001. These findings indicate enhanced functional integration in these regions in FAP. Refer to Table 2; Fig. 1A for details.

**Table 2:** Brain regions showing significant differences in FC with the left middle frontal gyrus as region of interest between FAP patients and HC (Alphasim-corrected *P* < 0.001).

Seed	Brain region	Peak MNI coordinates			t value	Cluster size (voxels, mm^3^)
		X	Y	Z		
The left middle frontal gyrus	Right middle frontal gyrus 1	45	54	18	5.95	359 (9,693)
	Right middle frontal gyrus 2	33	3	54	5.02	266 (7,182)
	Left superior temporal gyrus	− 54	− 6	− 3	4.81	177 (4,779)

Cluster size reported as: number of voxels (volume in mm^3^). Volume calculated as: number of voxels × voxel volume (27 mm^3^). Voxel size after normalization: 3.0 × 3.0 × 3.0 mm.

FAP: functional anorectal pain; FC: functional connectivity; HC: healthy controls; MNI: Montreal Neurological Institute.

### VMHC differences between groups

**Fig. 2:**
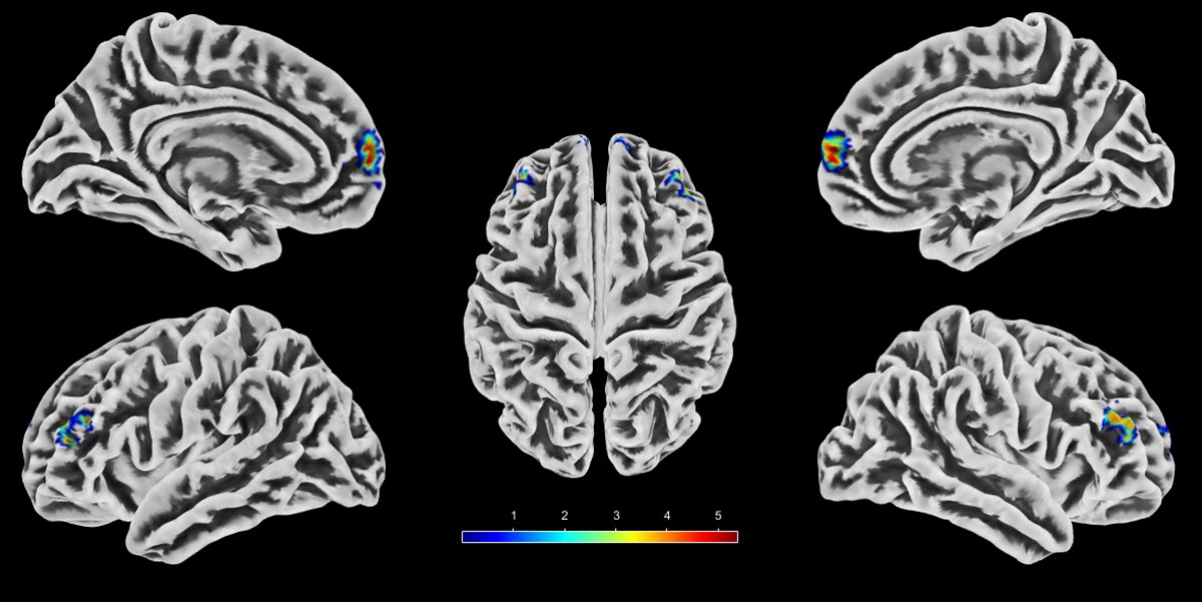
Voxel-mirrored homotopic connectivity (VMHC) alterations in functional anorectal pain (FAP). Regions showing increased VMHC in FAP patients versus HC (Alphasim-corrected *P* < 0.001). The color bar indicates *t*-values. Sagittal and axial views are shown.

As shown in Table 3; Fig. 2, compared with HC, FAP patients exhibited significantly increased VMHC in the MFG.L and left superior medial frontal gyrus (SFGmed.L) at Alphasim-corrected *P* < 0.001. No regions showed decreased VMHC in the patients relative to the controls.

**Table 3:** Brain regions showing significant differences in VMHC values between FAP patients and HC (Alphasim-corrected *P* < 0.001).

Cluster No.	Brain region	Peak MNI coordinates			t value	Cluster size (voxels, mm^3^)
		X	Y	Z		
Cluster 1	Middle frontal gyrus	− 45	48	21	4.45	37 (999)
Cluster 2	Superior medial frontal gyrus	− 3	63	6	5.88	28 (756)

Cluster size reported as: number of voxels (volume in mm^3^). Volume calculated as: number of voxels × voxel volume (27 mm^3^). Voxel size after normalization: 3.0 × 3.0 × 3.0 mm.

FAP: functional anorectal pain; HC: healthy controls; MNI, Montreal Neurological Institute; VMHC: voxel-mirrored homotopic connectivity.

### Correlation analysis results

in FAP patients, the enhanced FC between the MFG.L and one cluster of the MFG.R was negatively correlated with age (*R* = − 0.478, *P* = 0.009; Fig. 1b). This finding indicates that as patient age increases, the strength of this connection tends to decrease. No other correlations were detected between brain functional alterations and demographic or clinical variables (Supplementary Table S1 and Table S2 in the Supplementary Material.

## Discussion

Our study identified increased rs-FC in FAP patients, particularly between the MFG.L and regions such as the MFG.R and STG.L. Additionally, VMHC analysis suggested enhanced connectivity in the MFG.L and SFGmed.L compared with HC. These results suggest a pattern of heightened interhemispheric functional integration in FAP patients. Notably, the negative correlation between age and FC between the left and right MFG indicates that this heightened connection may diminish with age. Overall, these findings indicate alteration in interhemispheric communication in FAP patients, which may contribute to the complex pathophysiology of the syndrome.

The significantly enhanced rs-FC between the MFG.L and both the MFG.R and STG.L in FAP patients reflects a distinct neuroadaptive reorganization within prefrontal-executive and sensory-affective networks. This hyperconnectivity likely reflects compensatory top-down modulation by the DLPFC, which plays a critical role in cognitive control of pain and emotion regulation^30–32^. Specifically, heightened MFG.L-MFG.R connectivity may represent an effort to enhance interhemispheric coordination for inhibitory control over limbic hyperactivity (e.g., the amygdala) and maladaptive nociceptive signaling from the anorectal region^33,34^. This is consistent with established models of prefrontal engagement in chronic pain conditions, where MFG hyperactivity attempts to restore inhibitory balance in disrupted pain-processing networks^35–38^. Concurrently, the increased MFG.L-STG.L connectivity implicates dysregulated sensory-affective integration, as the STG processes visceral sensory input and assigns emotional valence to interoceptive signals^39,40^. This aligns with evidence from irritable bowel syndrome (IBS) research^41,42^, where MFG-STG hyperconnectivity correlates with visceral hypersensitivity and symptom severity, suggesting a shared neural substrate for central sensitization across functional gastrointestinal disorders involving aberrant attentional bias toward visceral stimuli^43^. Collectively, these FC alterations highlight an MFG-centered pathophysiology in FAP. Failed compensatory efforts to control pain via prefrontal networks coexist with amplified sensory-limbic processing, perpetuating the cycle of chronic pain and psychological distress characteristic of this disorder.

The heightened VMHC in the MFG.L and SFGmed.L in FAP patients corresponds with our prior findings of predominantly right-sided functional changes^8^. The elevated synchrony in the MFG.L/SFGmed.L may represent a futile compensatory attempt to regulate right-sided pain signals through top-down inhibition. Typically, the DLPFC plays a role in pain suppression via corticolimbic circuits. However, excessive interhemispheric coupling in FAP might reduce neural adaptability, contributing to central sensitization and psychological issues such as anxiety and depression. the elevated VMHC observed in the MFG.L and SFGmed.L may stem from impaired transcallosal inhibition^44^. GABAergic interneurons manage this inhibition, which normally maintains hemispheric functional segregation^45^. When this mechanism is compromised, it might result in excessive interaction between homotopic prefrontal regions in the two hemispheres^46^. This disruption may interfere with the normal role of each hemisphere in pain processing. For instance, the right hemisphere is typically dominant in sensory-discriminative pain processing, whereas the left hemisphere is more involved in cognitive-affective appraisal^47^. The over-synchronization between homotopic prefrontal regions might lead to unhealthy integration of visceral nociception and psychological distress, potentially amplifying maladaptive pain processing^48^. Importantly, the increased VMHC in the SFGmed.L, a key part of the DMN, hints at disrupted self-referential processing^49^. This might lead to a maladaptive focus on internal threats such as rectal discomfort, thereby intensifying symptom perception. This aligns with the clinical presentation of FAP, where psychological distress worsens visceral hypersensitivity and pelvic floor dysfunction^2,8^. The distinct pattern of VMHC increase in FAP highlights its unique neuropathology within the brain-gut axis, which differs from the classic “disconnection” models observed in chronic pain or psychiatric disorders^24,50^. Future research is needed to determine whether this VMHC pattern can serve as a biomarker for neuromodulation targeting the MFG.L and SFGmed.L to restore inhibitory balance.

The simultaneous increase of both FC and VMHC in FAP patients highlights distinctive frontal hyperactivity coupled with enhanced interhemispheric equilibrium. This pattern suggests a failure in transcallosal inhibition—a GABAergic-mediated process that normally maintains hemispheric segregation during sensory-affective processing^51,52^. In healthy brains, the right hemisphere predominantly processes sensory-discriminative aspects of pain (e.g., intensity and location), while the left prefrontal cortex regulates cognitive-affective appraisal (e.g., threat evaluation and emotional response)^53,54^. In FAP, however, the heightened FC between the MFG.L and MFG.R, alongside increased VMHC in the MFG.L and SFGmed.L, indicates pathological hyper-synchronization that disrupts this lateralization. This susceptibility amplifies right-hemisphere nociceptive signaling (e.g., from the anorectal region) while overloading left prefrontal attempts to exert top-down control, ultimately perpetuating pain chronification^53,55^. Our previous multimodal MRI study revealed decreased gray matter volume in the SFG.R, MFG.L, left middle temporal gyrus (MTG.L) and other brain regions^8^. These findings provide a critical context for the current functional observations: the left frontal FC/VMHC elevations likely represent a maladaptive compensatory response to right-sided nociceptive dominance. This asymmetric reorganization aligns with the clinical features of FAP, such as peripheral treatment resistance and comorbid psychological distress^2,3^. Such hemispheric dyscoordination exacerbates central sensitization through disrupted corticolimbic integration^56^.

The observed negative correlation between MFG.L-MFG.R FC strength and patient age reveal a critical decline in neuroplasticity in FAP, where the compensatory capacity of the brain diminishes with aging. Younger individuals may sustain stronger MFG.L-MFG.R connectivity to gate right-lateralized visceral pain processing, but this compensatory mechanism might decline with advancing age owing to reduced GABAergic neurotransmission and gray matter atrophy in dorsolateral prefrontal circuits^57–59^. Consequently, older FAP patients might exhibit heightened vulnerability to pain chronicity despite comparable psychological comorbidity severity, potentially explaining their poorer response to conventional therapies such as pelvic floor physical training. The absence of correlations between FC alterations and clinical pain/psychological scores further indicates that age is an independent modulator of prefrontal network efficiency, whereas FAP-related brain changes represent trait-like neural signatures rather than symptom-dependent fluctuations. Clinically, our integrated model suggests that cognitive-behavioral therapy (CBT), including mindfulness-based approaches, should be considered a foundational intervention for all FAP patients, given its established efficacy in managing chronic pain and associated psychological distress, and its evidence for inducing beneficial neurofunctional changes^60–62^. Building on this foundation, our findings tentatively suggest that treatment could be further stratified. Younger patients, who may possess greater neuroplastic potential, might derive particular benefit from the adjunctive use of neuromodulation techniques targeting the identified prefrontal circuitry to amplify therapeutic effects^63–65^. For older patients, or those who may not fully respond to first-line approaches, the combination of CBT with pharmacotherapy such as paroxetine might represent a particularly effective multimodal strategy^3,66,67^. These suggestions are hypothetical and intended to generate future research into personalized treatment algorithms for FAP. Collectively, these findings position age as a pivotal biomarker for precision therapeutics in FAP, suggesting that neural-resilience-adapted strategies might be used to restore disrupted interhemispheric homeostasis. Critically, the lack of correlation between neural alterations and clinical pain measures must be interpreted with caution. These findings may indicate that the observed interhemispheric hyperconnectivity is not a direct neural correlate of momentary pain perception. Instead, it might represent a trait-like feature of the broader FAP syndrome, reflecting a stable maladaptation in brain networks that underpins the complex interplay between chronic pain and affective comorbidity.

Taken together, our findings suggest that FAP involves aberrant interhemispheric hyperconnectivity. The heightened FC between the MFG.L and both MFG.R and STG.L, coupled with increased VMHC in the MFG.L and SFGmed.L, reveals maladaptive neural reorganization within prefrontal regulatory networks. This pattern suggests maladaptive overcompensation efforts to modulate right-lateralized nociceptive signaling through top-down inhibition, while simultaneously amplifying the sensory-affective processing of visceral stimuli. Critically, the age-dependent decline in MFG.L-MFG.R FC underscores eroding neuroplasticity in aging patients, explaining their poorer treatment response. These neural alterations represent trait-like signatures of FAP pathophysiology rather than symptom-dependent fluctuations, supporting their use as diagnostic biomarkers. The neural circuits identified in this study could potentially inform future therapeutic development, although this requires rigorous validation in interventional studies.

several limitations should be noted in this current study. Our findings should be interpreted considering methodological constraints. First, the moderate sample size (30 FAP patients vs. 21 HC) limits the statistical power for subgroup analyses such as age stratification and may affect generalizability. Second, the cross-sectional design precludes causal inferences about whether the observed FC/VMHC alterations drive or result from chronic pain. Therefore, the derivative discussion of potential clinical applications should be interpreted cautiously. Finally, recruitment exclusively from a Chinese population restricts the extrapolation of findings to other ethnic groups; validation in diverse populations is essential to confirm the cultural/biological generalizability of the identified interhemispheric imbalance pattern.

## Conclusion

This study establishes altered interhemispheric communication as a core mechanism in FAP, characterized by aberrant hyperconnectivity in the frontal networks. Key findings include heightened FC between the MFG.L and bilateral pain-processing regions, elevated homotopic synchrony in the medial prefrontal areas, and an age-dependent decline in interhemispheric FC. These alterations reflect maladaptive overcompensation to regulate right-lateralized nociception through impaired transcallosal inhibition. Our work positions FAP within a brain-gut axis dysregulation framework and suggests circuit-specific therapeutics to restore neural homeostasis.

## Supplementary Information

Below is the link to the electronic supplementary material.

Supplementary Material 1

## Data Availability

The datasets analyzed during this study are available from the corresponding author on reasonable request.
