# Supplementary material for: Enhanced interhemispheric functional connectivity in patients with functional anorectal pain
Source: Sci Rep. 2025 Nov 27;15:42489. doi: 10.1038/s41598-025-26490-3 (PMC12660719; doi:10.1038/s41598-025-26490-3)
Supplement: Supplementary file 1 — Supplementary Material 1 [file 41598_2025_26490_MOESM1_ESM.docx]

**Supplementary Table S1**. Results of correlation analyses between functional connectivity alterations and clinical variables in functional anorectal pain patients.

| **Seed** | **Brain Region** | **Correlation with Age, *R* (*P*)** | **Correlation with SF-MPQ, *R* (*P*)** | **Correlation with HAMD, *R* (*P*)** | **Correlation with HAMA, *R* (*P*)** | **Correlation with PSQI, *R* (*P*)** |
| --- | --- | --- | --- | --- | --- | --- |
| The left middle frontal gyrus | right middle frontal gyrus 1 | -0.236 (0.218) | 0.031 (0.872) | 0.069 (0.724) | 0.105 (0.587) | 0.283 (0.136) |
|  | right middle frontal gyrus 2 | -0.478 (0.009)* | -0.045 (0.818) | -0.008 (0.966) | 0.119 (0.537) | 0.276 (0.148) |
|  | Left superior temporal gyrus | -0.297 (0.117) | -0.092 (0.634) | 0.058 (0.765) | 0.314 (0.097) | 0.237 (0.216) |

*: *P*<0.05

HAMA, Hamilton Anxiety Scale; HAMD, Hamilton Depression Scale; PSQI, Pittsburgh Sleep Quality Index; SF-MPQ, short-form of McGill pain questionnaire.

**Table S2.** Results of correlation analyses between voxel-mirrored homotopic connectivity alterations and clinical variables in functional anorectal pain patients.

| **Cluster No.** | **Brain Region** | **Correlation with Age, *R* (*P*)** | **Correlation with SF-MPQ, *R* (*P*)** | **Correlation with HAMD, *R* (*P*)** | **Correlation with HAMA, *R* (*P*)** | **Correlation with PSQI, *R* (*P*)** |
| --- | --- | --- | --- | --- | --- | --- |
| 1 | middle frontal gyrus | -0.038 (0.845) | -0.103 (0.594) | -0.080 (0.679) | -0.041 (0.835) | 0.406 (0.029) |
| 2 | middle frontal gyrus | -0.147 (0.448) | -0.194 (0.313) | -0.254 (0.184) | 0.001 (0.994) | 0.110 (0.571) |

HAMA, Hamilton Anxiety Scale; HAMD, Hamilton Depression Scale; PSQI, Pittsburgh Sleep Quality Index; SF-MPQ, short-form of McGill pain questionnaire.
